# Dark Triad Traits and Sleep-Related Constructs: An Opinion Piece

**DOI:** 10.3389/fpsyg.2020.00505

**Published:** 2020-03-19

**Authors:** Kenneth Graham Drinkwater, Neil Dagnall, Andrew Denovan

**Affiliations:** Department of Psychology, Faculty of Health, Psychology and Social Care, Manchester Metropolitan University, Manchester, United Kingdom

**Keywords:** lucid dreaming, sleep-related constructs, dark triad traits, personality dimensions, methodological issues

## Introduction

Good quality sleep is vital to health and psychological functioning (see Steptoe et al., 2008). Indeed, insufficient sleep has a negative effect on chronic disease incidence and development (Perry et al., 2013). Alongside health implications, the investigation of sleep is important because it conceptually informs a range of academic disciplines (neuropsychology, physiology, psychology, etc.). Acknowledgment of these factors has stimulated research, which historically has identified sleep-related dissociative states (nightmares, dreams, etc.), and outlined factors that influence the timing, duration, and quality of sleep.

From a psychological perspective, investigators have placed great emphasis on examining relationships between sleep states, personality factors (e.g., Randler et al., 2017), and related constructs (e.g., creativity). However, relatively few studies have focused on lucid dreaming (LD) (e.g., Schredl et al., 2016).

LD is a distinct behavioral state characterized by awareness of dreaming during sleep, which involves the ability to control dream events, and/or purposefully awaken (Harb et al., 2016). Future individual differences research needs to consider LD since the phenomenon has important implications for models of human cognition. Explicitly, LD provides insights into the nature and constraints of consciousness, particularly the potential for reflective mindfulness (Kahan and LaBerge, 1994). Additionally, LD has useful applications for experiencers (solving waking problems, physical/mental healing, training motor skills, etc.) (Stumbrys and Erlacher, 2016), and possesses potential therapeutic benefits (e.g., reducing nightmare frequency) (Holzinger et al., 2015).

Even fewer studies in the domain of personality and individual differences research have examined relationships between LD and socially aversive traits (Marcus and Zeigler-Hill, 2015). This is an important research gap to bridge as interest in dark traits is ever increasing and related constructs (i.e., Machiavellianism, narcissism and psychopathy) possess characteristics, which are likely to affect lucid dreaming. In this context, the influence of the Dark Triad (DT) personality construct is fundamental (Paulhus and Williams, 2002). The recent emergence of work investigating associations between darker, social malevolent personality traits and variations in sleep-related behavior and states reflects this (e.g., Yang et al., 2019).

From this perspective, the DT is particularly important. The DT refers to three personality dimensions marked by manipulation and callousness: Machiavellianism, subclinical narcissism, and subclinical psychopathy (Jones and Paulhus, 2014). Machiavellianism denotes a calculative attitude encompassing the ability to control others, deception, self-centeredness and immorality. Although, individuals scoring high on Machiavellianism present as charming and impressive, these speciously “attractive” attributes mask propensity to hypocrisy, cynical worldview, and scheming.

Narcissism reflects a clash between grandiose identity and underlying insecurity that manifests as the need for constant ego-reinforcement (Jones and Paulhus, 2014). Several studies report the existence of two or more forms of narcissism (Miller et al., 2011). The most prevalent distinction being between grandiose and vulnerable. Grandiose comprises grandiosity, aggression and dominance, whereas vulnerable narcissism reflects a defensive and insecure grandiosity that obfuscates adverse cognitions, perceptions, and emotions (i.e., feelings of inadequacy, incompetence, and negative affect) (Miller et al., 2011).

Psychopathy indexes deficits in affect (i.e., callousness; disregard for others and lack of empathy) and self-control (i.e., impulsivity) (Hare, 1970; Cleckley, 1976; Lykken, 1995). Callousness is typically short-term. Hence, psychopaths lie for immediate rewards, even when this undermines their long-term goals (Paulhus and Williams, 2002). Thus, in the context of psychopathy, callous manipulation combines with immediate tendencies such as thrill seeking and recklessness to prompt related dispositions, and facilitate corresponding behaviors (Hare and Neumann, 2008). Authors often make a distinction between primary and secondary psychopaths. Historically, researchers have often linked primary psychopathy to genetic factors and secondary psychopathy to social factors (Skeem et al., 2007). Primary psychopaths are callous, calculating, manipulative, and deceitful, whereas secondary psychopaths share antisocial behaviors with primary psychopaths, but are remorseful and fearful (Sethi et al., 2018).

## Indicative Research

To explore further LD and individual differences, investigators need to consider the findings/scope of previous work. This has demonstrated that DT traits can influence sleep-related states/behavior and has produced theoretically important findings. For instance, Jonason et al. (2013) observed a link between “darker” DT elements (Machiavellianism, secondary psychopathy, and exploitive narcissism) and a night specialism (chronotype). This predisposes individuals toward optimal cognitive performance during the hours of darkness.

Additionally, Sabouri et al. (2016) found that Machiavellianism and psychopathy were associated with higher sleep disturbances, increased anxiety sensitivity, and greater intolerance of uncertainty. These outcomes aligned with previous research documenting relationships between negative affect and poor sleep (Whiteside and Lynam, 2001; Brand et al., 2016). Noting this, Sabouri et al. (2016) concluded that the association between DT traits and sleep disturbance arises from unfavorable cognitive–emotional processes. Specifically, rumination, poor coping strategies, and low emotion regulation. Relatedly, Yang et al. (2019) found Machiavellianism was directly associated with poor sleep quality, and indirectly associated via greater anger rumination. Additionally, primary and secondary psychopathy were indirectly associated with poor sleep quality via greater anger rumination. Secondary psychopathy had the strongest direct effect on poor sleep quality among the DT traits.

These findings were congruent with preceding studies reporting relationships between poor sleep, reduced emotion regulation (Brand et al., 2016), and lack of impulse control (Becker, 2014). In this context, LD may reduce negative emotions by allowing the dreamer to take control of the dream. Knowing that it is possible to govern dream content can facilitate the reduction of adverse affective content. Earlier work suggests that this can reduce distress within nightmares (Gavie and Revonsuo, 2010), and concomitantly lessen nightmare frequency and intensity, leading to better life quality during wakefulness (Soffer-Dudek, 2017).

## Discussion

Although studies examining relationships between sleep-related states and personality traits make important contributions to conceptual understanding of sleep, several methodological issues limit the generalizability of findings. For prospective research on LD and the DT to be effective, researchers need to acknowledge these concerns when designing studies, and discussing outcome implications.

A major limitation of previous work is that studies have typically used a cross-sectional method. This is where researchers collect data simultaneously, at one time point and/or within a brief duration (Levin, 2006). The cross-sectional method is criticized because responses represent only a “snapshot” of characteristics associated with the measured outcome at a particular point in time. Consequently, data provides only “estimates” of prevalence within populations. This explains why cross-sectional studies frequently provide limited correlation-based analysis and report weak correlations.

Even when researchers employ sophisticated analytical techniques, causation remains an issue. Particularly, it is difficult to conclude whether sleep-related experience/behavior derives from personality factors or causes enduring behaviors and perceptions. One potential remedy within mediation-based studies is reverse testing, where analysis compares the predicted model against an alternative. This statistically assesses whether the indirect effect of independent variable (X) on the dependent variable (Y) via the intervening factor (M) is significantly different from zero. Despite providing some indication of causality this approach is not always successful (Lemmer and Gollwitzer, 2017).

Another issue with cross-sectional studies is common method variance (CMV) (Chang et al., 2010). This denotes shared variance arising from the method used, rather than the constructs observed (Podsakoff et al., 2003). CMV creates false internal consistency, correlation arising from common context. This manifests as the tendency to respond consistently to unrelated items. Hence, one index of sleep may influence scores on another, or responses on DT factors. This is a major concern within sleep-related research because observed relationships are often weak, and CMV can inflate correlations (Lindell and Whitney, 2001). Thus, without safeguards there is an increased possibility of type 1 error. Studies can reduce the dangers of CMV by creating psychological distance between constructs, and by employing instructions that reduce social desirability effects and evaluation apprehension (Podsakoff et al., 2003). To guard against CMV, studies investigating relationships between LD and the DT should employ protocols that emphasize differences between constructs and response scales.

Furthermore, while repeated cross-sectional studies can enhance the reliability of findings, the cross-sectional approach still fails to control for the effects of unaccounted factors. One such variable, which investigators have frequently included in sleep-related research, is mental toughness. Mental toughness is a generic term that denotes enabling psychological resources that promote positive mental health and performance across a range of achievement contexts (Dagnall et al., 2019; Drinkwater et al., 2019). Noting this generality, Gucciardi (2017) defined mental toughness as “a state-like psychological resource that is purposeful, flexible, and efficient in nature for the enactment and maintenance of goal-directed pursuits” (p. 18).

Intervening constructs that influence relationships between sleep-related states and personality factors are problematic because they produce complex effects. For instance, greater mental toughness is associated with better sleep quality, shorter sleep onset latency, fewer awakenings, longer sleep duration, and reduced sleep complaints (Brand et al., 2014a,b). Additionally, higher levels of mental toughness correlate with positive psychological health outcomes (Gerber et al., 2018). Pertinently, mental toughness influences also DT traits. Particularly, Papageorgiou et al. (2019) observed that the subclinical narcissism to mental toughness pathway in their model predicted lower levels of psychiatric symptoms. Moreover, Papageorgiou et al. (2017) reported that mental toughness facilitated the development of the adaptive aspects of narcissism (e.g., coping behaviors).

Noting these factors, subsequent work on the relationship between LD and the DT should control for mental toughness, consider the role of moderating/mediating factors generally, and examine effects over extended periods using multiple time points. Although, multiple time point studies are prone to logistical difficulties (i.e., recruitment and retention) and expensive in terms of time and cost, they provide a nuanced understanding of how personality traits effect sleep-related measures over time (relationship stability).

Regarding LD, the use of standardized definitions and measurement indexes is vital to cross study comparisons. In the case of classifications, there exists significant variation across studies. For instance, while several use the Schredl and Erlacher (2004) conceptualization (e.g., Denis and Poerio, 2017), others employ different wording (e.g., Sestir et al., 2019), or have devised alternative measures (Aviram and Soffer-Dudek, 2018; the Frequency and Intensity Lucid Dream questionnaire, FILD).

Moreover, studies also index different aspects of LD. For instance, alongside prevalence and frequency (see Snyder and Gackenbach, 1988) papers often include measures of control and dream environment manipulation (Stumbrys and Erlacher, 2017). To ensure comparability it is important that researchers examining LD agree on standard operationalizations and indices. This is especially important when prior research is relatively limited as outcome variations resulting from different measurement instruments can produce conceptual fragmentation. This problem is not unique to LD. Indeed, work investigating other sleep-related states/behaviors draws on a range of measurement tools and indexes a variety of indicators. For example, there are multiple scales used to assess insomnia.

A further issue is that sleep-related studies regularly use self-report. Critics question the validity and accuracy of these because they assess psychological processes indirectly by drawing upon metacognitive insight and recall (Lance and Vandenberg, 2009). This leaves self-report measures vulnerable to subjective bias. Hence, it is advisable to corroborate LD self-report findings with objective indices.

Researchers examining the influence of the on sleep-related states/behavior need also to consider the role of DT sub-factors. Illustratively, Jonason et al. (2013) reported that only exploitive narcissism was associated with night specialism. Moreover, primary and secondary psychopathy have different relationships with anxiety-related constructs. Primary correlates negatively, whereas secondary is positively associated. This suggests that effects will vary as a function of DT factor type.

Finally, future studies need to establish cross-cultural, age and gender invariance. This will help to counter potential measurement bias. In the case of the Short Dark Triad (SD3, Jones and Paulhus, 2014), researchers report the instrument is invariant for language and culture (Pechorro et al., 2019). It is essential that investigators similarly evaluate sleep-related measures.

Addressing these issues will ensure that future research examining relationships between the DT and sleep-related measures generates more robust, convincing findings.

## Author Contributions

ND and AD: article development and composition. KD: draft review and creative oversight.

### Conflict of Interest

The authors declare that the research was conducted in the absence of any commercial or financial relationships that could be construed as a potential conflict of interest.
